# The Future of Pork Production in the World: Towards Sustainable, Welfare-Positive Systems

**DOI:** 10.3390/ani3020401

**Published:** 2013-05-15

**Authors:** John J. McGlone

**Affiliations:** Laboratory of Animal Behavior, Physiology and Welfare, Department of Animal and Food Sciences, Texas Tech University, Lubbock, TX 79409, USA; E-Mail: john.mcglone@ttu.edu

**Keywords:** sustainable, pigs, animal welfare

## Abstract

**Simple Summary:**

More pork is eaten in the world than any other meat. Making production systems and practices more sustainable will benefit the animals, the planet and people. A system is presented by which production practices are evaluated using a sustainability matrix. The matrix shows why some practices are more common in some countries and regions and the impediments to more sustainable systems. This method can be used to assess the sustainability of production practices in the future where objective, science-based information is presented alongside ethical and economic information to make the most informed decisions. Finally, this paper points to current pork production practices that are more and less sustainable.

**Abstract:**

Among land animals, more pork is eaten in the world than any other meat. The earth holds about one billion pigs who deliver over 100 mmt of pork to people for consumption. Systems of pork production changed from a forest-based to pasture-based to dirt lots and finally into specially-designed buildings. The world pork industry is variable and complex not just in production methods but in economics and cultural value. A systematic analysis of pork industry sustainability was performed. Sustainable production methods are considered at three levels using three examples in this paper: production system, penning system and for a production practice. A sustainability matrix was provided for each example. In a comparison of indoor *vs.* outdoor systems, the food safety/zoonoses concerns make current outdoor systems unsustainable. The choice of keeping pregnant sows in group pens or individual crates is complex in that the outcome of a sustainability assessment leads to the conclusion that group penning is more sustainable in the EU and certain USA states, but the individual crate is currently more sustainable in other USA states, Asia and Latin America. A comparison of conventional physical castration with immunological castration shows that the less-common immunological castration method is more sustainable (for a number of reasons). This paper provides a method to assess the sustainability of production systems and practices that take into account the best available science, human perception and culture, animal welfare, the environment, food safety, worker health and safety, and economics (including the cost of production and solving world hunger). This tool can be used in countries and regions where the table values of a sustainability matrix change based on local conditions. The sustainability matrix can be used to assess current systems and predict improved systems of the future.

## 1. Introduction

More pork is eaten in the work than any other terrestrial meat ([1], Table 1). About 37% of all meat consumed in the world is pork (110 million metric tonnes, mmt)—ahead of beef (67 mmt) and chicken (104 mmt). The Food and Agriculture Organization (FAO) of the United Nations (UN) lists livestock production as industrialized (meaning indoors with a waste containment system), grazing or mixed. In 2011, China had about half of the world pork production (50 mmt), and the USA was second largest in numbers of pigs (10 mmt) with only one-fifth as many pigs as China. Germany was third in pork production with half the USA level (5 mmt). The EU as a whole is a significant pork producer. Over 80% of the world’s pork is produced in Asia, the EU and North America. About 7.4 mmt of pork was traded in 2011 (6.7% of the total)—thus most pigs are currently produced in the country in which they are consumed, although this may change with increases in labor and land costs in developed countries. 

**Table 1:** Meat amount and % consumed in the world [1].

Meat	Meat consumed, 2012 (mmt)	Percentage of Meat (%)
Pork/Porcine	110.8	37.4%
Poultry	104.5	35.3%
Bovine	66.8	22.6%
Ovine	13.9	4.7%
**Total common meats**	296.0	100.0%

Population of the world is increasing slowly, but it is expected to plateau in the next 30–50 years. Growth in the population increases the demand for pork (and other meats). Furthermore, as developing countries become more affluent, the population consumes more meat. Thus, while world population may reach 50% higher than today, world meat consumption will likely double in the next 30–50 years. Industrialized pork production facilities depreciate over a 20–30 year period and would be replaced at the end of a depreciation cycle with new and improved facilities. Thus, in the next decades, not only will pig and pork facilities double in size, they will be completely replaced as a part of the normal turnover of assets. The newer systems are expected to be more sustainable, although we are unsure what exactly that means at this time. Here I speculate on how pork production systems will become more sustainable. Certainly, the first requirement of a sustainable system is that it must be economically competitive. 

While pork production of 100 years ago was mixed and grazing, today all developed countries use the industrialized systems for the majority of pigs and pork produced. Developing countries are rapidly moving pork production to the industrialized model. But whether the system is industrialized or grazing, is each system sustainable? Will it protect and nurture the animals, the environment and the people, or will the production system be negative towards animals, people or the environment? This remains to be seen. 

The modern scientific literature tends to be studies from Europe and North America and hence are European-centric (assuming the Americas are an outcrop of Europe), but we must realize that more than half the pork produced and consumed is from/in Asia and China in particular. Asian pork production is rapidly changing to become more like the European/American model, changing from grazing and mixed (backyard) systems to the industrialized model. A new world view of pork production should focus on Asia and the European/American models. Pork industry growth will also continue in Eastern Europe and Latin America. North American facilities are fairly new and will be turned over in 10 to 30 years and at that time, what passes as a sustainable system may be different than today. We have an opportunity to shape the production system whenever a region develops newer farms. We have this opportunity now with pork production in Asia and Eastern Europe because it is growing steadily. For Western Europe and North America, the pork industry is not rapidly growing at the moment. Pig farms will turn over its assets in 10 to 30 years and so changing the system now would be costly. But in a developing region, new farms can be built in a more sustainable fashion—or mistakes can be made building old-style farms that will take decades to modify. What is needed is a system to determine which systems are sustainable in an objective and transparent manner. Here I apply the animal welfare matrix to develop matrices of sustainability. The beauty of the matrix is that it may be changed by its user to reflect its current and anticipated future situation. Even the weighting of the matrix can be changed to be made more accurate by a given user. This approach is not new [2], except as applied to the issue of sustainability of pork production practices.

Knowing that current and future farms ought to be more sustainable is intuitive and self-evident. The argument comes down to what is meant to be sustainable. We would mostly agree that a sustainable farm will be in business a very long time and will not harm the people, animals, and community and planet, and it will nurture and mesh with the local ecosystem (on both micro and macro levels). Agricultural enterprises are simultaneously a way of life and a business. To be sustainable, a farm must be economically viable. 

I argued to a displeased EAAP audience in 2000 that animal welfare should be considered within the context of a sustainable production system [3]. The view is now more main stream that animal welfare improvements should be checked against other society issues. We should check animal welfare improvement against concerns such as environmental pollution (soil, water and air pollution, including the farm’s carbon footprint), food safety, economics, worker health and safety, population changes, cost of food, world hunger and market forces. Furthermore, we should not promote systems that may improve animal welfare but cause issues with other society issues (e.g., food safety, environmental issues, worker health and safety, world hunger, and the nutritional value of the meat produced). One can see that it would be undesirable to use a system that provides improved animal welfare but introduces bacteria into the meat or increases pollution. 

With all of this as background, we have a serious issue with a growing and changing world population. Our need for pork is likely to significantly increase as the world has world population increases, as rates of poverty decline, and as people eat more meat, including pork. 

The world currently supports about one billion pigs/year. How would pork production look if there were two billion pigs given the other constraints of land and resources? We have two choices as we develop more sustainable systems; either change the systems we have or build new systems. Changing the current systems would be likely to be cost prohibitive, unless the changes are minimal. 

It is less problematic to build new facilities that are more sustainable than previous systems. This is how change happens normally. For example, pigs were mostly kept outdoors a hundred years ago. Then, indoor systems were developed. The systems of today are better than those of 50 years ago because they protect pigs from weather extremes and are able to be sanitized, but cost little as building strategies have likewise evolved and improved. 

We should not underestimate the power of the media and society to change production practices. Government rules can change practices (such as the ban of sow gestation crates in Europe and some USA states). But market forces can provide a more powerful and timely pace of change. For example, several EU and USA retailers have issued statements requiring group housing of sows, well ahead of legislative actions (more on this later). 

Below I will discuss three example animal welfare issues. The same approach could be used for other pig industry animal welfare issues. These three issues span the breath of different issues and are currently the subject of legislation or market actions. The discussed issues span from production system, to penning types to a production practice. 

To facilitate a systematic approach to these issues, a sustainability matrix was employed to assess the overall sustainability of each practice. The idea of applying an ethical matrix to animal welfare is not new [3,4]. Here I broaden the concept to develop an ethical matrix for sustainability. Some ethical matrices become complex and use defined weighting [5]. Here I present the matrices but allow the reader to adjust the numbers based on new information or on their own values (ethical, scientific understanding or others). Seeing where the science leads us for a given issue is only one piece of the puzzle. We must also consider ethical values, economics and even an emotional view of practices and procedures. Although some scientists consider emotions the antithesis of their view, one must accept that part of consumer purchasing behavior is based on emotion. In this model, the emotional view of consumers is one factor in animal welfare perception. Each animal product has an animal welfare perception and that perception is based on the understanding (or lack of understanding) of the purchaser of the production system and practices. While an up-scale grocery store may sell pork from “free-range” pigs, the consumer does not expect this product feature among conventional pork products. 

Each production practice and systems ought to be evaluated for its sustainability. This happens over time naturally. But if we undergo the exercise ahead of time and act on our objective understanding of hard facts and economic, ethical and emotional views, we could save much time, money and even prevent animal and human suffering. The specific aim of this paper is to outline a mechanism to evaluate the sustainability of modern pork product today and in the future using three diverse examples ranging from the entire system to a production practice. This model explains why systems are as they are today and the model proposes a positive way forward in the development of sustainable pork production systems, including attention to pig welfare.

## 2. Example #1: Outdoor *vs.* Indoor Production

Here I use the term “indoor” production rather than the more negative term “industrialized.” The language we use, as scientists, ought to be as objective as possible. The term industrialized implies something more machine-like rather than nurturing animals. Ruth Harrison’s Animal Machines [6] was so named at least in part due to the negative, non-empathetic impression that words like “machine” and “factory farming” conjure up when talking about an animal system. In starting a public movement, such emotional terms serve a purpose. Here, however, the intended presentation is to highlight the benefits and issues with indoor and outdoor production in an objective manner. 

Animals can be nurtured in either indoor or outdoor systems. They can also be treated poorly in either system. In this example, the assumption is that we are comparing well-designed and well-managed systems (indoor and outdoor), based on the highest ethical and scientific standards within economic constraints of commercial production. 

One can present an outdoor system in a positive light by showing green pastures, sunshine and spacious fields [7]. However, in many climates the outdoor pigs experience cold and snow, rain and mud, heat, and extreme weather. A complete analysis should include all seasons. Outdoor pigs can easily experience internal and external parasites. 

One can present an indoor system in a positive light by showing sanitized pens, groups of clean, healthy pigs fed nutritious diets [8]. However, some indoor units can be dirty, dark, and have high ammonia and unhealthy pigs. 

When well-managed systems are compared, the public perception of the outdoor system is better than the perception of the indoor system (Table 2). Organic systems require access to the outdoors because of a perception that the meat would be, in some way, better. We know that the pork from an outdoor system has about the same general eating quality as pork from an indoor system [9,10]. Pigs from the outdoor system have different muscle fiber types [9,10] but this does not change the pork tenderness or flavor. Interestingly, although consumers blind to the housing system cannot tell indoor from outdoor pork taste, when they saw a label, they rated pork from an outdoor system as more desirable in eating quality than indoor-raised pork [11]. Thus, human perception, on average, rates the outdoor system more desirable than the indoor system.

Many studies have compared the welfare of pigs raised indoors and outdoors. Stress hormone levels, health, mortality, and other production measures, on average, do not differ between indoor and outdoor-reared pigs [12,13]. Outdoor pigs in many regions require anti-parasitic medicine due to infestation of fields with internal and external parasites. Enteric diseases can be an issue for outdoor pigs as well. Conversely, respiratory (and perhaps) enteric diseases are easily spread among indoor-kept pigs. Thus, the health challenges for indoor and outdoor pig production are about equal overall. Either system can be managed with healthy pigs on clean land or in clean buildings with medications to control some diseases.

Specific welfare issues are found among indoor and outdoor pigs. Concrete slatted flooring can injure sows’ feet. Outdoor sow require their toe nails trimmed when outdoors to prevent them from having long toe nails that cause lameness (trimming sow toe nails is nearly a lost art). Outdoor sows may consume poisonous plants or sharp objects. However, they spend more time rooting and digging with their snouts than indoor pigs [14,15]. Confining indoor grouped sows to small spaces in expensive facilities may increase aggression. While this has not been directly studied, we have noticed little or no scars associated with fighting among outdoor sows probably because the submissive sow can escape attack when they have a pasture as opposed to an indoor pen. 

**Table 2:** Comparisons of Outdoor and Indoor production systems in a sustainability matrix, with the indoor, industrialized system as a baseline *.

Issue	Indoor	Outdoor **
**Animal welfare perception**	0	10
**Animal welfare science**	0	0
**Productivity & Economics**	0	0
**Climate variability**	0	−2
**Pork quality (real and perceived)**	0	2
**Environmental impact**	0	0
**Worker health and safety**	0	−5
**Community interface**	0	0
**Food safety (*Salmonella*)**	0	−20
**Zoonotic diseases (Influenza risk)**	0	−5
**Total**		−20

* Here we assume that both indoor and outdoor systems are well-designed and operated systems. The indoor system is the most common system in developed countries. A negative total indicates the alternative to the standard is less acceptable than the standard; a positive total would mean the alternative is preferred. Table values inform areas in which improvement may be needed in a component of sustainability. ** General consumer perception is better for outdoor production by a large factor, especially to uninformed consumers who look only at the system in the best conditions. Climate variability that causes variation in production and welfare results in a negative value. Pork quality may be better, although the science gives it only a small advantage, while consumers favor use of the outdoor system, generally. Worker health and safety is problematic in the outdoor system currently. And food safety and zoonotic concerns represent large negative values for the outdoor system.

The environmental impacts of indoor and outdoor production are about the same for well-managed systems. Indoor systems collect manure (feces, urine and wasted water and feed) in liquid/slurry form or in a more solid form if bedding is used. Outdoor pigs distribute manure by walking around [16]. If fed the same, pigs in indoor and outdoor systems produce the same amount of manure. However, if an indoor system uses a manure lagoon or pond, then the manure will decompose and move into the air rather than being deposited in the soil. Manure directly deposited in the soil will have some volatilization into greenhouse gases, but these values have not been determined. However, we can speculate at this time that the amount of greenhouse gases that move into the atmosphere is likely to be greater when a manure lagoon is used than when the manure is spread by animals over the land. The direct land application of manure by walking, moving animals is determined by many factors including especially the vegetative ground cover (the more plants, the more nutrient absorption can take place) and the animal density (if animals are too dense, manure nutrients can run off the field). 

The indoor system can use any number of systems to capture the manure nutrients and minimize environmental (water, soil and air) pollution. Manure can be dried and/or composted, or used in a methane digester to generate energy, for example. 

The outdoor system can use green pastures and rotate animals to minimize environmental pollution. Using best management practices would never or rarely have outdoor pigs on barren land (no vegetation) but this system is difficult to achieve in some climates. 

Economics do not differ between indoor and outdoor systems. The main determinant of economic success in modern pork production is the scale of production. Large farms have lower costs per animal than smaller farms. If an outdoor system was built on the same scale as modern indoor systems, the costs of production would be about the same [17]. 

Worker health and safety is more challenging in the outdoor system. There is more walking, running, lifting and risk of injury in the outdoor system than the indoor system. This could be changed with research and refinement of the system. For example, on a commercial farm near Texas Tech University that had both indoor and outdoor systems, the number of injuries and deaths (e.g., lightning strike or tractor accidents) in the outdoor system was much higher than their indoor system.

Communities like the economic value of commercial animal agriculture. Farms provide jobs and money for the local economy. If the systems are both well-managed, they are likely to have a good relationship with the local community. School children will visit the indoor or outdoor systems and interact with the animals. Local leaders will value the economic impact of an animal production unit. If the unit has an offensive odor or pollutes the water or is otherwise unpleasant, then the community will not be a supporter of the farm.

Zoonotic diseases fall in two categories: those that might be in the food and those that might infect people from live pigs. The particular set of infectious diseases that might be found in pork are bacteria such as *Salmonella*, *E. coli*, *Listeria*, *Campylobacter*, among other enteric diseases shared by pigs and people. While all of these have not been studied in indoor and outdoor production systems, there is literature on *Salmonella*. Indoor and outdoor pigs have *Salmonella* at times. In our studies, our indoor pigs were not shedding *Salmonella*. However, the outdoor herd had *Salmonella* in the wallow water, feed and in the sows [18]. We learned that wild birds continually inoculated the outdoor environment with *Salmonella* [18]. Wild birds also have a risk of carrying strains of Influenza that may infect pigs and people. The risk of food safety and Influenza has virtually shut down outdoor pig production in some areas. In this case, zoonotic risk blows up the model for outdoor pig production meaning outdoor pig production using is not sustainable. If the zoonotic risks were eliminated, outdoor pig production would be favored based on the below sustainability model compared with raising pigs in the indoor system. However, managing extremes of weather and worker health and safety issues could also be improved in the outdoor system.

## 3. Example #2: Gestation Sow Housing Systems: Crates *vs.* Pens

Gestation sow housing systems are the subject of animal welfare concerns in Europe and North America. There is little concern in other countries at this time, but because international trade in pork is growing and animal welfare is expected to be a part of trade agreements, animal welfare is expected to be an international issue. 

The EU has to have its sows out of gestation crates by 2013 [19]. In the USA, some individual states have banned the gestation crate, but the states with the most commercial sows have not banned the crate at this time. The sow gestation crate is the most visible animal welfare issue in commercial pork production in the USA at this time. So is the gestation crate sustainable?

Clearly, the public perception about the gestation crate is negative in Europe and the USA. Both conservative and liberal USA states have banned the gestation crate (e.g., Arizona and California). The margins have been consistently about 2 to 1 that American people voted to ban the individual gestation crate. Thus, public perception opposition to the individual gestation crate transcends political and other segments of American society. In the EU, the more Northern and Western countries banned the individual gestation crates before the entire EU banned the system.

Over 30 USA retail firms that sell pork have issued statements that they will source pork from farms that do not use the gestation crate (McDonald’s, Burger King, Safeway, *etc.*). Two retail firms (Domino’s and Bob Evans) have said they will use science to determine their pork supply production practices and will therefore allow pork from sows in gestation crates. Thus, market forces are pushing USA pork producers away from the gestation crate. 

The sustainability matrix is simpler for sow gestation system than it was for indoor *vs.* outdoor systems. Many variables are the same for the two systems, including environmental impact, zoonoses, and community interactions. Considering animal welfare science, we have a very interesting situation. The totality of the science, as reviewed by four groups over time [13,20,21,22] suggests that the welfare, on average, does not differ however, one EU review [23] concluded the welfare of sows in gestation crates was lower than when they are in group housing. 

What is important here is that in a few decades, pig facilities will change in location and probably style. If one were to build a new farm in China or the EU/North America today, for example, would they put in individual gestation crates or group pens? New farms or remodeled farms are putting in individual crates at the moment in Asia and Latin America and group pens in the EU and in the Americas. The bottom line of a sustainability matrix related to gestation sow housing would clearly be different, at the moment, in China or Europe. Economics wins in the absence of an animal-welfare trump card. A trump card was delivered by national or regional legislation in the EU and by state laws or market preferences in the USA (but not a national law). A law or market action is required for any sustainability issue to trump economics. Markets can require a certain sow housing system (like group pens rather than crates during gestation) and then the market pays for this change. Without a direct market demand (and paying farmers more for product), only a government law can give animal industry incentive to change in the face of adverse economics. 

Iowa is a USA state that has a large number of sows and no law banning gestation crates. One can argue that the most sustainable position for an Iowa pork producer is to keep sows in gestation crates to avoid the economic penalty. Conversely, a pork producer in Denmark building a new facility would be wise to put in group housing because of the EU rule and thus, in Denmark, the group gestation system is the most sustainable today.

One concept is that sustainable refers to the long term. But one must get past short time economic forces before a long term approach can be taken. While it is certain to this observer that the world will not have gestation crates in the future, the rate of their demise in less developed countries is unclear and unpredictable.

Two situations are presented in Table 3 relative to the world standard among indoor production. The standard is the gestation crate (it is the most common in the world). In one column, the gestation crate is against the law as in the EU and some USA states. In another column, the gestation crate is not illegal and economics forces its use if farmers want to be competitive. In one case, the gestation crate is not sustainable because of legal issues if the crate was to be used. In the other case, economics would make a farmer less competitive and at risk of bankruptcy if they use a group housing system that requires more floor space. One cannot predict the future with certainty—if the world enters into a negative economic downturn and meat cost becomes the driving force in production, then the gestation crate is more sustainable. But if the world’s population continues to increase income as in China today, then meat consumption will increase and people may be willing to pay a bit more for pork raised in a manner consistent with people’s ethics (although the ethical view of Asians may not be the same as Western people—this remains to be seen). If markets demand pork without the use of gestation crates and they do not pay more for it at first, then when farmers go out of business, the price of pork from the surviving farmers will cost a little more based on the laws of supply and demand. It would be better for farmers, of course, if the consumer paid more for pork with higher standards, and did not force the markets to force people with individual crates to go out of business. 

**Table 3:** Comparison of indoor sow gestation systems: well-managed pen *vs.* crate using a sustainability matrix.

Issue	Individual Crate	Group Pen China, Brazil, Iowa **	Group Pen * EU or California **
Animal welfare perception	0	10	10
Animal welfare science	0	0	0
Productivity & Economics *	0	−11	−9
Environmental impact	0	0	0
Worker health and safety	0	0	0
Community interface	0	0	0
Food safety & Zoonoses	0	0	0
Total	0	−1	+1

* The value of economics changes from −11 to −9 if either market forces or government rules force adoption of group housing of sows during gestation. In the EU and some USA states, the gestation crate is banned and thus there is no economic value to use of the gestation crate. The current assessment of sustainability of gestation sow keeping (individual crate *vs.* group pen) depends on the location of the farm. ** The animal welfare perception is clearly better for group pens than for keeping sows in crates both in geographies where the crate was banned and for other regions of Europe and North America. While productivity is about equal for sows in crates and group pens, economics favors individual crates since they require less floor space. Other factors are about equal for the two systems (crates *vs.* groups) regardless of the geography and legal issues.

## 4. Example #3: Male Pig Castration

Farm animals experience a number of painful procedures that are termed “standard agricultural practices” [24]. Within pig farming, these commonly include tail docking, iron (or other medication) injection, and castration. Some less common procedures include teeth trimming, toe nail trimming, boar tusk trimming and ear notching. 

Castration is one procedure that is in the suite of painful procedures that are called litter processing [25]. Among these procedures, physical castration elicits the greatest stress response in terms of increased cortisol [26] and pig stress vocalizations [27]. Here I discuss the sustainability of castration only. Other procedures should also be addressed for their sustainability in the future. Castration is used as the example here because (a) it may be the most painful of the standard agricultural practices, (b) there are alternatives, and (c) it is the subject of EU regulation and activist attention. 

The most common method of castration of pigs is to physically castrate males (at 2–5 days of age) without the use of pain relief. The procedure is like a surgical procedure, but without sterile instruments or a sterile field or a dedicated surgical suite. Pigs are typically castrated in the room where they were born or in an adjacent hallway. A knife or sharp instrument is used to cut the scrotum and underlying membranes and the testicle is manually pulled either completely out of the pig or the testis is pulled and the spermatic cord is cut or serrated. The scrotum and the spermatic cord have nerves and nerve endings that signal pain when stretched or cut. 

Pigs are castrated for one primary reason (to reduce boar taint) and one secondary reason (to reduce pig aggression). The primary reason is to eliminate boar taint which is a natural pheromone used in male courtship. Male pigs produce this pheromone in their saliva, urine and fat that has an offensive odor [28]. This boar pheromone is 5-α-androsten-16-en-3-one (and related steroids). Adrostenone has an off odor that is exacerbated when heated (or when pork is cooked). Steroid hormones cause the synthesis of androstenone and an off-smelling fecal compound called skatole. Androstenone and skatole combine to cause bad-smelling pork called “boar taint.” Androstenone and skatole are significantly reduced in adult male pigs that have been castrated [29]. Physical castration reduces the rate of “boar taint” to less than 5% of male pork carcasses (a level equal to or lower than among physically castrated males). 

The best way to assess the sustainability of castration is to compare physical castration (the most common method of castration in the world) with alternatives to physical castration. Alternatives include the following options.

Market at a younger age. In the UK, meat pigs are marketed at a younger age than, for example, in the USA. Intact males convert feed to pork more efficiently which will help world hunger, lower production costs and reduce the environmental impact since there are fewer manure nutrients than when males are castrated [30,31]. The idea is that if male pigs are slaughtered before puberty, then they will not have boar taint. However, onset of puberty (and therefore boar taint in the meat) is quite variable. The percentage of male pigs with boar taint increases with age. At the same time, the economics of pork production holds that the system is more cost efficient if pigs are processed at a heavier weight. After all, the labor and other costs to process a 100 kg carcass is nearly the same as a 50 kg carcass—and you get more meat with a larger carcass. Larger carcasses spread the cost of processing over more kg of pork, thereby lowering the cost of pork. Processing plants drive farmers to produce larger carcasses. Many male carcasses over 80 kg will have boar taint. A 65 kg carcass would reduce the risk of boar taint—but most developed countries and many developing countries market pigs at a body weight that would generate a carcass over 80 kg. To have those industries lower slaughter weights to prevent boar taint would be very costly (how costly is not exactly known) and the price of retail pork would have to increase. Poor people would consume less pork. But there would be an environmental advantage because male pigs are more efficient at converting grain to meat than castrated males. Presently, in all but a few countries, the economics of pork processing trumps the animal welfare and environmental benefits of marketing pigs at a younger age. 

Genetic selection. Scientists and farmers have proposed selecting against boar taint [31,32]. One can reduce the level of boar taint by genetic selection; however, genetic lines of pigs are not currently available with low boar taint at current marketing weights in most countries. 

Physical castration with pain relief. One can physically castrate pigs and give medications to reduce the pain. If the pain was relieved, there may still be an ethical issue with physical removal of the gonads. However, the negative consequences of the option of pain relief are four-fold: (1) pain medications are not currently approved by regulatory bodies in pigs, and (2) studies on efficacy of pain relief medications have not identified medications that totally relieve this particular pain, (3) this option adds cost and a risk of pig injury or mortality with no added economic benefit, and (4) some consumers have an aversion to anything not natural being administered to a food animal. 

Castration pain falls in two phases: during the procedure and after the procedure as the wound heals. Administration of intra-scrotal and intra-testes lidocaine (a local anesthetic) before castration eliminated the pain-induced behavioral effects of castration [33]. However, healing takes several days and the local anesthetic wears off in 30–60 minutes [34]. The post-procedural pain is not relieved by local anesthetic [35]. People have used analgesics to attempt to relieve the procedural pain and the post-procedural pain. Procedural pain was not relieved by analgesics evaluated so far [34]. Topical local anesthetics are easy to administer, but they do little to blunt the procedural pain of physical castration [35].

Immunological castration. For over a decade, a product has been on the market in some countries (over 60 countries now) that immunologically castrates male pigs. This immunization binds the pigs’ natural GnRH (a reproductive hormone) and temporarily reduces steroid levels in the blood. Consequently, boar taint is virtually eliminated in immunologically castrated male pigs. Feed efficiency benefits of the male pig are captured [29], which provides an added environmental benefit [30]. The intact males are given the immunization late in finishing in time to reduce boar taint and capture the benefits of using intact males. This option has a payback that is reported to outweigh its cost [36]. Consumers may object to anything unnatural injected in a pig (as they might likewise feel about anesthetics). Much has been written about consumer concerns about human safety of pork from immunologically castrated males (more-so in some countries than others). Where it was assessed, consumers preferred immunological castration over castration without pain relief [37]. 

Table 4 presents a sustainability matrix for conventional physical castration with and without pain relief and immunological castration. For countries and regions that market at a lighter weight, this may be the preferred option; however for the majority of the world, marketing at a lighter weight is not economically feasible. Genetic selection may help reduce the rate of boar taint in markets that market at a lighter weight, but genetic lines are not currently available that have low boar taint levels for heavy weight pigs. Pain relief, although only partially efficacious, is not a viable option because most countries’ regulatory bodies have not approved effective analgesics and anesthetics in pigs. 

Clearly, Table 4 will change with added technologies. However, at the moment, for the majority of pigs in the world, only two options are available. Immunological castration (IC) removes the need for physical castration (PC). The animal welfare concern with PC is therefore eliminated when IC technology is used. And the science supports this view (see above). Producers have to pay for IC technology, but it returns money to the producer making it economically positive. The environmental impact is also improved because intact males convert feed more efficiently than PC males early in life. Worker health and safety is negative because of the unlikely risk of self administration of the drug by workers and because of the need to make injections which itself holds a small risk of needle stick. Some consumers do not appreciate technologies like vaccination against a hormone and so the food safety item is slightly negative for IC males. However, the overall sustainability of IC is better than using PC technique based on this analysis. 

**Table 4:** Comparison of selected methods of castration using a sustainability matrix. This table applies to countries where pigs are marketed at relatively heavy weights (over 110 kg live weight).

Issue	Physical Castration (PC)	Immunological Castration (IC) *
Animal welfare perception	0	2 *
Animal welfare science	0	2
Productivity & Economics	0	1
Environmental impact	0	2
Worker health and safety	0	−1
Community interface	0	0
Food safety & Zoonoses	0	−1 *
Total	0	5

* The animal welfare perception and food safety are in two areas. Not physically castrating pigs is viewed positively by knowledgeable consumers, however there is a fear about any technology that is injected in the animal (fear of contaminated food that may harm a person, even if evidence is presented to indicate otherwise). IC is better for pig welfare both based on science and human perception. Economics favor IC over PC. The environmental impact is less for IC than PC. Worker health and safety is a concern in case humans get injected with the immunogen (although safety measures are in place). This table would indicate IC would be preferred to PC unless the fear of contaminated food is stronger than the negative reaction to PC without pain relief (normally, the fear of contamination with biotech products is less than the negative view of PC without pain relief).

## 5. Conclusions

The user may put more or less value in some cells in each sustainability table and the bottom line decision will change. Each table value may vary with geographies and cultures. Each table is provided as a starting point for discussion. Having a logical way to assess technologies is positive and beneficial to both consumers and to agriculture.

One of the main pivot points in assessing sustainability is the economic competitiveness of any production system, penning system, or procedure on the farm. If a farming system is not economically competitive, it cannot survive. 

In the sow housing debate, in practice in most of the world, the science holds that economics trumps perception of animal welfare. Thus, the individual sow gestation crate is the most common system found in the world of indoor pig production. But legislation trumps economics in some countries and USA states. Therefore, we find quite different sow systems in different countries and even regions within countries. One could argue that sustainability should not be this way, that one sow system should be the most sustainable. But at the moment, this is not the case. It is impossible to predict if the world will have a single best most sustainable gestation sow penning/crating system over time. This difficulty arises because, for the sow gestation keeping situation, to provide a perceived animal welfare improvement (group pen instead of individual crate) has a real economic cost (group penning requires more space). For gestation sow housing, economics and perceived welfare are at cross purposes. 

For pig castration, the research is clear that castration is a painful body alteration. Providing pain relief is costly and not currently efficacious. Immunological castration is an alternative that is more sustainable. However, consumers who fear a vaccine of a hormone analog, there may be a perceived issue. This is a grave concern. The basic concern is a fear of technology. It will only be through advanced technology that we can keep food animals healthy and more productive. And only with advanced technologies can more people be fed with the feed resources we have (in limited supply). Educating consumers about more advanced technologies may avert fears of new technology and allow more people to be able to afford higher quality foods. 

Finally, science can be used to identify more sustainable systems of pig farming and pork production. But science must be considered within the context of human emotion and economics to obtain a glimpse of which systems and practices are the most sustainable. 
